# Mortality Among Japanese With a History of Kawasaki Disease: Results at the End of 2009

**DOI:** 10.2188/jea.JE20130048

**Published:** 2013-11-05

**Authors:** Yosikazu Nakamura, Eiko Aso, Mayumi Yashiro, Satoshi Tsuboi, Takao Kojo, Yasuko Aoyama, Kazuhiko Kotani, Ritei Uehara, Hiroshi Yanagawa

**Affiliations:** Department of Public Health, Jichi Medical University, Shimotsuke, Tochigi, Japan; 自治医科大学公衆衛生学教室

**Keywords:** mucocutaneous lymph node syndrome, long-term prognosis, mortality rate, follow-up, Japan

## Abstract

**Background:**

The long-term outcomes of Kawasaki disease (KD) are unknown.

**Methods:**

Fifty-two collaborating hospitals collected data on all patients who had received a new definite diagnosis of KD between July 1982 and December 1992. Patients were followed until December 31, 2009 or death. Standardized mortality ratios (SMRs) were calculated based on Japanese vital statistics data.

**Results:**

Of the 6576 patients enrolled, 46 (35 males and 11 females) died (SMR: 1.00; 95% CI: 0.73–1.34). Among persons without cardiac sequelae, SMRs were not high after the acute phase of KD (SMR: 0.65; 95% CI: 0.41–0.96). Among persons with cardiac sequelae, 13 males and 1 female died during the observation period (SMR: 1.86; 95% CI: 1.02–3.13).

**Conclusions:**

In this cohort, the mortality rate among Japanese with cardiac sequelae due to KD was significantly higher than that of the general population. In contrast, the rates for males and females without sequelae were not elevated.

## Abstract (ja)

**背景：:**

川崎病既往者の長期予後はいまだに明らかではない。

**方法：:**

1982年７月から1992年12月の間に52の協力病院は川崎病確実例の診断を受けた全ての患者のデータを収集した。患者は2009年12月31日または死亡まで追跡された。人口動態統計をもとにした標準化死亡比（SMR）を計算した。

**結果：:**

観察された6576人のうち，46人(男35人、女11人)が死亡し、SMRは1.00（95信頼区間［CI］：0.73-1.34)であった。急性期の高いSMRに対して、急性期以降の全対象者の死亡率は高くなかった。心後遺症を持たない群での急性期以降のSMRは0.65（95％CI：0.41-0.96)であったのに対して、観察期間中に心後遺症を持つ13人の男と１人の女が死亡し、SMRは1.86（95％CI：1.02-3.13)であった。

**結論：:**

このコホート集団では川崎病による心後遺症を持つ群の死亡率は一般集団よりも高かった。一方、心後遺症を持たない群では男女とも死亡率は上昇していなかった。

## INTRODUCTION

A serious concern in Kawasaki disease (KD) is cardiac sequelae, including coronary aneurysms, coronary stenosis, and valvular lesions.^1^ In addition, vasculitis—the main characteristic of KD—could potentially lead to rapid progression of atherosclerosis in children, as their cardiovascular system is immature. However, evidence for this hypothesis is inconsistent.^2^^–^^12^

Follow-up of persons with a history of KD is important in preventing chronic diseases in adulthood. Rapid progression of atherosclerosis among those with a history of KD would suggest that such a history is a risk factor for cardiovascular and cerebrovascular diseases. Therefore, if a history of KD is found to be an additional risk factor, persons with such a history may need more-intensive control for other risk factors, such as blood pressure, serum lipids, and smoking habits.

A number of follow-up studies of KD patients have been conducted, but many only monitored patients who continued to visit hospitals.^13^^–^^17^ However, many such patients were under observation because they had known cardiac problems. Although these studies are useful in revealing KD outcomes, they cannot help in determining the prognoses of persons without cardiac sequelae. To determine the outcomes of persons with a history of KD, the present study used data collected in a follow-up study of patients with KD who visited any of 52 hospitals in Japan during the acute phase of KD.^18^^–^^24^ This article presents the results (until the end of 2009) of the eighth follow-up examination of the cohort, which comprises patients who visited any of the 52 hospitals between July 1982 and December 1992.

## METHODS

The methods of the follow-up examination and the persons assessed were nearly the same as those described in the report of the previous follow-up examination.^24^

### Inclusion criteria

In 5 nationwide surveys of KD in Japan, 8417 patients who visited hospitals during the period from July 1982 through December 1992 were reported by the 52 collaborating hospitals. From these potential cohort members, the following were excluded: 652 patients without a definite diagnosis of KD, according to the diagnostic guidelines established by the Japan Kawasaki Disease Research Committee,^25^ 384 cases of recurrent KD, 786 patients presenting on or after the 15th day of illness, and 19 foreign nationals. Thus, only patients who were Japanese nationals, had received a definite diagnosis of KD, and were treated within 14 days of onset were included in this study. A definite case was defined as a patient presenting with at least 5 of the 6 major symptoms of KD, ie, fever, bilateral conjunctival congestion, changes in the lips and oral cavity, polymorphous exanthema, changes in the peripheral extremities, and acute nonpurulent cervical lymphadenopathy^25^ or with 4 of these symptoms plus cardiac lesions such as coronary aneurysms.^25^ The decision to include only patients presenting at hospitals within 14 days from disease onset was intended to avoid bias from including patients who were treated at large referral hospitals for late cardiac sequelae. Foreign nationals were excluded because their resident registration system differs from that for Japanese citizens and because data on foreign nationals are not available for follow-up. Ultimately, 6576 patients were enrolled in our study.

### Protocol

The members of the cohort group had been followed from the time they first presented at a hospital for treatment of KD until the end of December 2009 or death, whichever occurred first. For all members, except those whose deaths were known through previous observations, the *koseki* system (permanent resident registration system)^26^ was checked at municipal offices in 2010. It is possible for a person to enroll in this system anywhere in the country, even if he/she lives abroad. Being registered in the system indicated that the patient was alive on January 1, 2010. If a patient had died before the last day of 2009, the *koseki* system was used to obtain information on his/her death. In cases of death, a copy of the death certificate was obtained from local officers of the District Legal Affairs Bureaus of the Ministry of Justice, to determine cause of death. Official approval to use these records was obtained from the Civil Affairs Bureau of the Japanese Ministry of Justice.

### Statistical analysis

Duration of observation for each patient was calculated and stratified by sex, age (by month during the first year of life, by year during the period from age 1–4 years, and by 5-year age group thereafter), and calendar year. Expected number of deaths was calculated by multiplying the observation time for each patient by the death rate that was calculated using data from the Japanese vital statistics for each group, also stratified by sex, age, and calendar year. Standardized mortality ratios (SMRs), ie, the ratios of observed to expected numbers of deaths, were examined, after stratification by sex, illness phase (acute phase [within 2 months after the first visit to a hospital] and thereafter), and presence of cardiac sequelae after the acute phase. In addition, cause-specific SMRs were examined for diseases associated with relatively high numbers of deaths, namely, malignant neoplasms (International Classification of Diseases, Ninth Revision [ICD-9] codes 140–208, International Classification of Diseases, Tenth Revision [ICD-10] codes C00–C97); external deaths (ICD-9 codes 800–999, ICD-10 codes S00–T98); accidents (ICD-9 codes E800–E949, ICD-10 codes V01–X59); and suicide (ICD-9 codes E950–E959, ICD-10 codes X60–X84). In the Japanese nationwide surveys of KD that were conducted when follow-up started, cardiac sequelae were defined as presence of dilatation (including aneurysms), stenosis, occlusion of a coronary artery and/or myocardial infarction, or a valvular lesion at 1 month after KD onset.^27^ On the basis of a Poisson distribution,^28^ an exact 95% CI was calculated for each SMR. An SMR with a 95% CI that did not include unity was considered to be statistically significant.

### Ethical approval

This study was reviewed and approved by the Jichi Medical University Institutional Review Board (No. Eki-10-16, June 28, 2010).

## RESULTS

The sex and age distributions of the members of the cohort group at KD onset were consistent with the epidemiologic features of KD in Japan.^24^^,^^29^ On December 31, 2009, the age range of the cohort was 17 to 39 years. The age and sex distribution on that day is shown in Table 1. Before that day, there were 46 deaths, 10 of which are included in the current follow-up. We could not verify the vital status of 30 persons after January 1, 2010, and they were thus classified as lost to follow-up in the current observation (although their observed person-years until the end of the observation were added to the total observed person-years). Ultimately, the follow-up rate was (6576 − 30)/6576, or 99.5%. Of the 6576 patients, 1003 (15.3%, 649 males and 354 females) were reported to the nationwide surveys as cases of cardiac sequelae.^24^ This proportion did not differ markedly from that in the overall KD patient population in Japan.^29^

**Table 1.** Age distribution at end of follow-up (December 31, 2009)

Age (years)	Males	Females	Total
15–19	336 (8.9)	232 (8.2)	568 (8.6)
20–24	1497 (39.8)	1120 (39.8)	2617 (39.8)
25–29	1644 (43.7)	1274 (45.2)	2918 (44.4)
30–34	221 (5.9)	159 (5.6)	380 (5.8)
35–39	10 (0.3)	7 (0.2)	17 (0.3)

Deaths	35 (0.9)	11 (0.4)	46 (0.7)
Lost to follow-up	16 (0.4)	14 (0.5)	30 (0.5)

Total	3759 (100)	2817 (100)	6576 (100)

Percentages in parentheses.

Due to rounding, percentages may not sum to 100%.

The number of observed person-years was 148 295 overall, 84 595 for males, and 63 700 for females; therefore, the average observation periods for all members, males, and females were 22.6, 22.5, and 22.6 years, respectively.

The causes of death for the 46 fatal cases are shown in Table 2. There were 29 internal deaths and 17 external deaths. Among the internal deaths, the most frequent cause of death was KD, followed by malignant neoplasms. Two patients died of heart disease, but KD was not mentioned on the death certificates. One was a boy who developed KD at age 4 years 3 months, which was followed by cardiac sequelae and died of acute myocardial infarction due to right coronary aneurysms at age 17 years 4 months. The other was a boy who developed KD at age 5 months, followed by cardiac sequelae and died of acute heart failure at age 19 years 3 months. The external deaths included 7 accidents, 7 suicides, and 2 homicides (ie, murder–suicides in which parents killed their child and then themselves). One patient, for whom the cause of death was unknown, died from an overdose of an antipsychotic agent. Investigators were unable to determine whether the death was an accident or suicide.

**Table 2.** Cause of death for 46 patients with a history of Kawasaki disease (KD)

Cause of death	No.	Remarks
All	46	

Internal death	29	
KD	11	
Heart disease	2	No mention of KD on death certificates
Congenital anomalies of the circulatory system	4	
Malignant neoplasms	7	
Other diseases	5	

External death	17	
Accidental injuries	7	
Suicide	7	
Homicide	2	
Unknown	1	Overdose of antipsychotic agent (accidental death or suicide)

Table 3 shows numbers of deaths and SMRs for 8 subgroups, by sex. Among all cohort members, 35 males and 11 females died. The SMRs were approximately 1.0 for both sexes and were not statistically significant. The deviations were not significant in either group. Although the overall mortality rate was not elevated after the acute phase of KD, it was significantly higher after the acute phase among those with cardiac sequelae; the SMRs for patients with cardiac sequelae were 1.86 overall and 2.27 for males. The SMRs for malignant neoplasms were also elevated, especially among males, but not significantly. The numbers of deaths due to accidents and suicides were relatively large, but the SMRs were lower than unity.

**Table 3.** Numbers of observed and expected deaths, and standardized mortality ratios (SMRs)

Disease phase	Cause of death	Cardiac sequelae	Sex	No. of deaths		SMR (95% CI)

				Observed	Expected	
all	all	all	both	46	45.6	1.00 (0.73–1.34)
			male	35	32.0	1.09 (0.76–1.52)
			female	11	13.6	0.81 (0.40–1.45)

after acute phase	all	all	both	38	44.7	0.85 (0.60–1.17)
			male	29	31.4	0.92 (0.62–1.33)
			female	9	13.2	0.68 (0.31–1.29)

after acute phase	all	with sequelae	both	14	7.5	1.86 (1.02–3.13)
			male	13	5.7	2.27 (1.21–3.87)
			female	1	1.8	0.56 (0.01–3.14)

after acute phase	all	without sequelae	both	24	37.2	0.65 (0.41–0.96)
			male	16	25.7	0.62 (0.36–1.01)
			female	8	11.5	0.70 (0.30–1.37)

all	neoplasms	all	both	7	5.0	1.41 (0.56–2.89)
			male	5	3.1	1.60 (0.52–3.73)
			female	2	1.9	1.07 (0.13–3.87)

all	external deaths	all	both	17	25.1	0.68 (0.39–1.08)
			male	14	18.9	0.74 (0.40–1.24)
			female	3	6.2	0.48 (0.10–1.41)

all	accidental injuries	all	both	7	14.0	0.50 (0.20–1.03)
			male	7	10.9	0.64 (0.26–1.32)
			female	0	3.1	0.00 (0.00–1.17)

all	suicide	all	both	7	9.5	0.73 (0.30–1.52)
			male	6	6.9	0.87 (0.32–1.89)
			female	1	2.6	0.39 (0.01–2.17)

Table 4 shows the details of the 14 patients with cardiac sequelae who died, according to interval from KD onset to death. Patient 2 in Table 4 drowned during swimming; however, because of the lack of autopsy findings, it was unclear whether death was due to simple drowning or from drowning due to heart failure. A boy who developed KD at age 4 years 3 months, which was followed by cardiac sequelae, died from acute myocardial infarction (patient 6). Although KD was not mentioned on the death certificate, it was strongly suspected to be the ultimate cause. A man (patient 10) died of (suspected) acute heart failure during cardiopulmonary arrest but could not be resuscitated in an emergency unit. Although there was no mention of KD on the death certificate, death due to KD was strongly suspected. If these ambiguous cases are classified as related to KD, 7 deaths were related to KD (patients 1, 2, 3, 5, 6, 8, and 10), and 7 were not (patients 4, 7, 9, 11, 12, 13, and 14).

**Table 4.** List of 14 deaths among patients with cardiac sequelae due to Kawasaki disease (KD)

Sex	Age		Interval between KD onset and death	Cause of death

	At onset	At death		
M	1y3m	2y3m	11 months	Coronary artery insufficiency due to KD
M	8m	8y9m	6 years	Sudden death while swimming
M	3y5m	9y5m	6 years	Coronary artery insufficiency due to KD
M	5y2m	13y2m	7 years	Pneumonia due to flu
M	2y9m	15y7m	12 years	Acute MI due to KD
M	4y3m	17y4m	13 years	Acute MI
M	9y3m	22y4m	13 years	Suicide
M	10m	13y10m	13 years	Acute MI due to KD
M	2y0m	17y6m	15 years	Suicide
M	5m	19y3m	18 years	Acute heart failure (suspected)
M	1y5m	24y6m	23 years	Osteosarcoma
M	3y11m	27y6m	23 years	Suicide
M	2y9m	27y3m	24 years	Squamous cell carcinoma of the lung
F	8m	26y8m	25 years	Overdose of antipsychotic agent

MI: myocardial infarction.

## DISCUSSION

The current study revealed that mortality rates among patients with a history of KD in Japan were not higher than those in the vital statistics for the country. Although mortality rates were lower among persons without cardiac sequelae, they were higher for those with cardiac sequelae.

Fourteen deaths were observed among cohort members with cardiac sequelae due to KD, as shown in Table 4. As mentioned above, 4 deaths were known to be caused by KD (patients 1, 3, 5, and 8 in Table 4), and 3 deaths were suspected to be due to KD, although the disease was not mentioned on the relevant death certificates (patients 2, 6, and 10). The other 7 deaths were internal deaths due to causes other than KD or external deaths. As shown in Table 3, the expected numbers of deaths among those with cardiac sequelae was 7.5; thus, there were 6.5 (14 − 7.5) excess deaths. This value is very close to the present number of deaths due to KD (including the 3 suspected cases). Therefore, we believe that our data are consistent.

Our findings suggest that cardiac sequelae are more severe in males than in females, as 13 of the 14 deaths among persons with cardiac sequelae were among males. Giant coronary artery aneurysms, and cardiac sequelae in general, are more common among males than among females.^30^ Although the reasons are unclear, male patients with KD appear to be more vulnerable to vasculitis due to KD.

In Japan, physicians (pediatric cardiologists and others) are greatly interested in loss to follow-up among patients with cardiac sequelae due to KD. At the 32nd Annual Scientific Meeting of the Japanese Society of Kawasaki Disease, in October 2012, 1 section focused on this problem. Our data show that mortality among those with cardiac sequelae due to KD was higher than that among the general population. Therefore, all such persons should be managed by cardiologists or pediatric cardiologists.

It remains to be confirmed whether atherosclerosis progresses more rapidly among those who developed vasculitis during childhood, when the circulation system is immature.^31^ If vasculitis accelerates atherosclerosis, mortality rates for cardiovascular disease and cerebrovascular disease among the subjects of this study would likely be elevated. However, as shown in Table 1, about 85% of the observed population were in their 20s and thus not at high risk for such diseases. To determine the ultimate effects of vasculitis, we must continue follow-up.

As shown in Table 3, the SMRs for external deaths—in particular those for accidental injuries—were low, although not significantly. The reason for this finding is unknown, and it may be a chance result. Continued follow-up of this cohort may also shed additional light on this finding.

This study has important strengths. First, it is an epidemiologic study and not an animal or *in vitro* experiment. Therefore, external validity is high when the results are applied to other persons with a history of KD. Second, the follow-up rate was high, 99.5%. Low follow-up rates introduce selection bias. Use of the *koseki*, or permanent-resident registration system, in Japan for follow-up of participants ensures that data on vital status are exact. Because death certificates are used to ascertain cause of death both in this study and in vital statistics, the data in Table 3 are comparable.

Some limitations also exist. First, although the follow-up rate was high, it was not 100%. Selection bias would be a concern if some patients dropped out because of serious health problems. Second, cause of death was ascertained only by using death certificates, not from clinical data. In addition, we observed only the end-point, death, and have no information on participant lifestyles; therefore, we could not evaluate participant quality of life, such as limitations in activities of daily living due to periodic hospital visits. Third, the control group, ie, the general population in the vital statistics, includes not only those with a history of KD but also the participants in the current study. However, this is not a serious concern because the bias is toward the null, which means that the observed SMRs greater than 1.0 were underestimated, namely, that the actual SMRs are higher than those we observed. Finally, the young age of the cohort members did not permit us to answer the study question, that is, whether a history of KD is a risk factor for cardiovascular and cerebrovascular diseases. Therefore, follow-up of this cohort should continue.

### Conclusions

This long-term follow-up study of KD patients showed that although the mortality rate was higher among those with cardiac sequelae due to KD than among the general population, it was not higher among those without sequelae. This cohort should continue to be monitored to determine if a history of KD is a risk factor for atherosclerosis.

## ONLINE ONLY MATERIALS

Abstract in Japanese.
