# Supplementary material for: Mortality Among Japanese With a History of Kawasaki Disease: Results at the End of 2009
Source: J Epidemiol. 2013 Nov 5;23(6):429–34. doi: 10.2188/jea.JE20130048 (PMC3834280; doi:10.2188/jea.JE20130048)
Supplement: Abstract in Japanese. [file je-23-429-s001.pdf]

日本における川崎病既往者の死亡率：２００９年末までの結果

中村好一、阿相栄子、屋代真弓、坪井聡、古城隆雄、青山泰子、小谷和彦、上原里程、柳川洋（自治医科大学公衆衛生学教室）

背景：川崎病既往者の長期予後はいまだに明らかではない。

方法：1982 年 7 月から 1992 年 12 月の間に 52 の協力病院は川崎病確実例の診断を受けた全ての患者のデータを収集した。患者は 2009 年 12 月 31 日または死亡まで追跡された。人口動態統計をもとにした標準化死亡比（SMR）を計算した。

結果：観察された 6576 人のうち，46 人（男 35 人、女 11 人）が死亡し、SMR は 1.00（95 信頼区間［CI］：0.73-1.34）であった。急性期の高い SMR に対して、急性期以降の全対象者の死亡率は高くなかった。心後遺症を持たない群での急性期以降の SMR は 0.65（95%CI：0.41-0.96）であったのに対して、観察期間中に心後遺症を持つ 13 人の男と 1 人の女が死亡し、SMR は 1.86（95%CI：1.02-3.13）であった。

結論：このコホート集団では川崎病による心後遺症を持つ群の死亡率は一般集団よりも高かった。一方、心後遺症を持たない群では男女とも死亡率は上昇していなかった。

キーワード：川崎病、長期予後、死亡率、追跡，日本
